# Skills Required in the Care of Cancer Patients Who Undergo Surgery in the Hospital-Home Transition

**DOI:** 10.1007/s13187-021-01964-w

**Published:** 2021-01-27

**Authors:** Gloria Mabel Carrillo, Mónica Liliana Mesa, Daira Vanesa Burbano

**Affiliations:** 1 Department of Nursing, Faculty of Nursing, National University of Colombia, Street 30 No, 45-01 Bogotá, Colombia; 2 Group of Nursing, Instituto Nacional de Cancerología, Bogotá, Colombia; 3 Department of Nursing, Faculty of Nursing, National University of Colombia, Bogotá, Colombia

**Keywords:** Education, Cancer, Patient discharge, Surgery

## Abstract

It is essential to recognize what care is required by patients undergoing surgery in the transition from hospital to home to provide guidance for plans for release and follow-up and to achieve patient adherence to these plans. The objective of this study is to describe the skills required for the care of cancer patients who undergo surgery after hospital discharge. An exploratory-type descriptive approach was adopted, including 290 cancer patients who underwent surgery at a reference center in Bogotá, Colombia. Hospital discharge was followed by 4 weeks of telephone follow-up to investigate the skills required for care on the basis of the CUIDAR tool. The participants had a mean age of 59.3 years, with the majority being female and having low levels of education. The most prevalent type of cancer found was breast cancer, followed by colon and rectal, prostate, stomach, cervical, lung, and ovarian cancer. The first follow-up identified needs for care in most of the CUIDAR dimensions, predominantly instrumentation, knowledge, and anticipation. The fourth follow-up, which found reduced needs, focused on knowledge of diet and eating, physical activity, the management of sadness and anxiety, a permanent telephone hotline, and sharing with loved ones. Cancer patients who underwent surgery require skills for at-home care that need to be addressed in hospital discharge programs and with structured telephone follow-up. Telephone follow-up interventions need to be consolidated in hospital release or hospital discharge programs that address these care needs.

## Introduction

According to the World Health Organization (WHO) [1], cancer is the second leading cause of death in the world, and there has been an increase in incidence in middle- and low-income countries that has elicited global alarm in recent years. Cancer is a complex disease involving long periods of time and generally requiring high levels of care, which is perhaps why the need to offer patients safe, continuous, and comprehensive care from one day to the next has been increasingly emphasized.

Oncological surgery represents one of the primary treatments for breast, esophageal, stomach, liver, pancreatic, colon, rectal, and skin cancer as well as melanoma, sarcoma, and other cancer types. Surgery is considered a cornerstone in all phases of the disease, i.e., as a diagnostic used to assess the extent of neoplasia, as a treatment intended to be curative, as palliation, and as therapy to treat complications and alleviate associated sequelae [1].

How the subject and his or her family experience surgical recovery will depend on many factors, including the disease being treated, the specific type of intervention being performed, the general state of health, and any existing comorbidities. To understand the magnitude and scope of these surgical interventions, the patient will require education and training in self-management regarding the administration of medications, a catheter and other invasive devices, dietary adaptation, mobility, and the management of symptoms such as pain, nausea, weakness, and insomnia, among other aspects. The patient will need to adapt to the long-term side effects, which will depend on the type of surgery performed [2].

With the purpose of addressing the intervention needs required by patients in the Colombian context and taking Meleis’ transitions theory as our point of departure [3], we consolidated the CUIDAR (*competencia para el cuidado en el hogar*, in Spanish) strategy, theoretically defined as the capacity, ability, and preparation of a person to be able to take care of him- or herself at home. According to Meleis, certain transitions must be included in the practice of nursing, such as those related to the health and illness processes. A transition is considered healthy according to indexes of progress and results. A fundamental component is the development of an efficient relationship between the patient and the nurse.

For the health team, it is essential to recognize what the care needs are that users have during this hospital-home transition to guide the discharge and follow-up plans and to achieve patient adherence to them, considering the problems and typical situation experienced after discharge. In this regard, the competency for home healthcare (Spanish: *competencia para el cuidado en el hogar*, or CUIDAR) [4, 5] has been studied as a tool that measures from 6 components, the aspects required for an adequate transition from hospital to home by people with chronic disease and their family caregivers.

By conducting telephone follow-ups after the discharge of cancer patients who underwent oncological surgery, the team proposed identifying what the care needs was that they reported according to the 6 dimensions of home care competency and constituting those needs as key elements to be included in the hospital discharge plans.

## Aim

The primary aim was to describe the home care competency needs of cancer patients who underwent oncological surgery after their hospital discharge through follow-up calls.

## Methods

### Design

This study takes a quantitative, descriptive, and exploratory-type approach through telephone follow-up. It is a secondary project of a protocol approved by the Ethics and Research Committee of the National Cancer Institute (Bogotá, Colombia). This study was conducted from November 2016 to May 2018.

The data were collected by a questionnaire survey.

### Setting and Sample

A convenience sampling method was used to enlist participants. The data were collected by a questionnaire survey. Participants were recruited between January and November 2017 from the National Cancer Institute in Bogotá, the capital city of Colombia.

The inclusion criteria were as follows: (1) adults diagnosed with cancer who were in the postoperative period of cancer surgery; (2) who were older than 18 years of age; and (3) who had telephone access to receive calls during discharge.

Out of 325 potential patients, only 312 were linked to the study; however, only 290 completed the four telephone calls, for a response rate of 89.23%. The reasons for withdrawal from the study were as follows: the lack of time to answer calls (12 cases), hospital readmission (6 cases), and no desire to continue (4 cases).

A list of scheduled surgeries and hospital releases was prepared each week, with intentional assignment to the study performed by verifying that the participants met the inclusion criteria and wished to voluntarily participate.

### Ethical Considerations

Ethical considerations included the ethical guidelines for biomedical research prepared by the Council for International Organizations of Medical Sciences (CIOMS) [6] and the parameters established in Resolution 8430 of 1993 issued by the Ministry of Health of Colombia [7]. Consideration was given to aspects related to informed consent, voluntary participation, and the handling of confidential information.

### Data Collection

All patients included in the study received weekly telephone calls for a 4-week period after their hospital release, and the calls were conducted by nursing professionals.

The nurses gave general instructions to the patients to take into account before discharge, and during the phone calls, they provided information and education according to the needs indicated by the patients.

Each call lasted approximately 20 min. During this time, care needs were explored by each home care competency dimension, and information and instruction on skills management were provided. The same competency dimension was applied for home care in each telephone call intervention.

### Measures

#### Patient Characterization

Items explored patient aspects regarding age, gender, level of education, marital status, occupation, socioeconomic status, medical diagnosis, and the surgical intervention performed. The length of hospital stay was not a variable explored in the study.

#### CUIDAR—Follow-Up After Discharge

This measure was developed by the research group on the basis of the instrument of skill in at-home care, defined as a person’s capacity, ability, and preparation to deliver home care. It includes 6 categories that are indicated by the Spanish acronym CUIDAR [5] (thus making it easy to remember): *conocimiento* (knowledge), *unicidad* (uniqueness; personal conditions), *instrumental* (instrumentation), *disfrutar* (enjoyment; well-being), *anticipación* (anticipation), and *relación social e interacción* (relationships and social interaction). During each telephone follow-up session, the care needs were ascertained on the basis of these categories.

### Data Analysis

The data were analyzed with the SPSS version 22.0 package. Continuous variables were expressed as the mean and standard deviation, and categorical variables were expressed as percentages.

## Results

### Patient Characteristics

The 290 patients had an average age of 59.3 years, with a standard deviation (SD) of 12.9 years. The majority of patients were female and had low levels of education, a married marital status, the occupation of homemaker, and a low socioeconomic status. Most reported low levels of dependence and an intact mental state (Table 1).

**Table 1:** Socio-demographic characterization of the sample

Variables		
Age		59.3±12.9
Gender (%)	Male	40.7
	Female	59.3
Mental state (%)	Intact	99.3
	Slight alteration	0.7
Level of schooling (%)	Low	77.9
	Medium	6.2
	High	12.4
	None	3.4
Marital status (%)	Single	24.8
	Married	57.9
	Separated	10.3
	Widowed	6.9
Occupation (%)	Homemaker	51.0
	Employee	6.9
	Independent work	32.4
	Pensioner	9.0
Socioeconomic stratum (%)	Low	22.1
	Medium	72.4
	High	5.5

Source: Research data (2018)

**Fig. 1:**
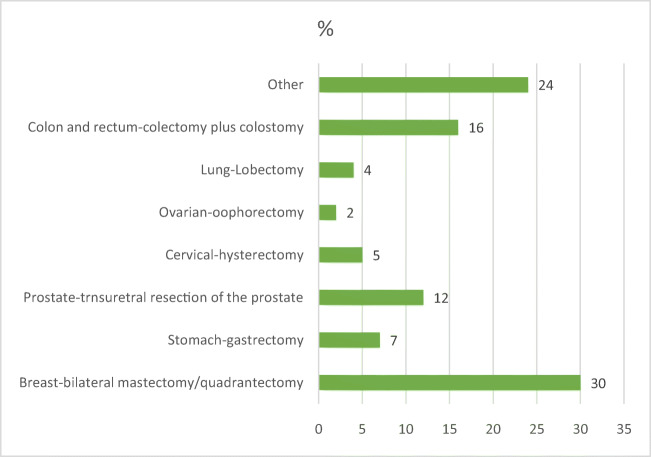
Type of cancer and surgical intervention of the participants. Source: Research data (2018)

The most prevalent type of cancer found was breast cancer, followed by colon and rectal, prostate, stomach, cervical, lung, and ovarian cancer. In terms of the type of surgical intervention, bilateral mastectomy/quadrantectomy ranked first, followed by colectomy and then colostomy, transurethral resection of the prostate, gastrectomy, hysterectomy, lobectomy, and oophorectomy, consistent with the cancer diagnosis (Fig. 1).

### Competency Needs for Care (CUIDAR)

During the first follow-up, the team identified needs for care in most of the CUIDAR dimensions, predominantly instrumentation, knowledge, and anticipation. The following were the most relevant aspects for the participants: guidance on the application of anticoagulants, techniques for pain management, physical activity, care with colostomy bags and barriers, the management of drainage, knowledge about diet and nutrition, constipation management, the detection of signs and symptoms of surgical wound infection, the availability of a permanent telephone emergency line, the administrative management of procedures and appointment authorization, and the need to share with their loved ones.

In the second follow-up, the needs related to the knowledge and instrumentation dimensions decreased; however, the need related to physical activity increased. In the anticipation dimension, the need for guidance on the signs and symptoms of surgical wound infection increased, as did the need for administrative procedures for the authorization of procedures and appointments, with the latter being significant. An increase in the relationship component was noted, specifically the need to share with loved ones.

In the third follow-up, it is worth highlighting the increase in the need for guidance on physical activity in the instrumentation dimension. Albeit at smaller percentages, the needs related to administrative management for the authorization of procedures and appointments, the permanent telephone service line, the signs and symptoms of surgical wound infection persist, as do the needs related to sadness and anxiety management (enjoyment dimension) and sharing with loved ones (relationship dimension).

**Fig. 2:**
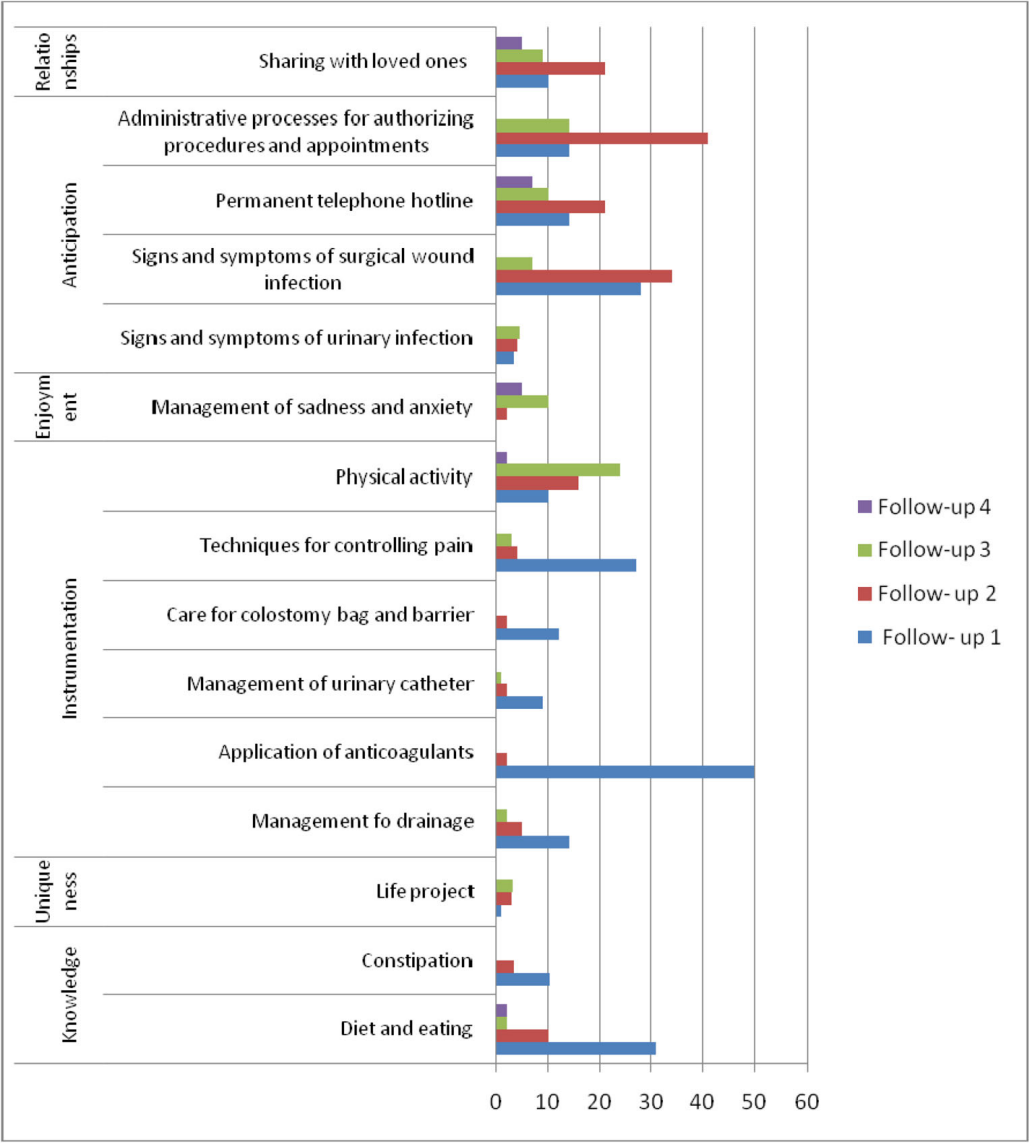
Skills required for at-home care of patients who have undergone surgery in each follow-up. Source: Research data, (2018)

In the fourth follow-up, the needs indicated were lower and focused on knowledge of diet and food, physical activity, the management of sadness and anxiety, the permanent telephone attention line, and sharing with loved ones (Table 2) (Fig. 2).

**Table 2:** Skills required for care of patients who have undergone surgery in each follow-up

Skill	Need	Follow-up 1 *n* (%)	Follow-up 2 *n* (%)	Follow-up 3 *n* (%)	Follow-up 4 *n* (%)
Knowledge	Diet and eating	50 (31)	30 (10)	5 (2)	5 (2)
	Constipation	30 (10.3)	10 (3.4)	0	0
Uniqueness	Life plan	3 (1)	8 (2.8)	9 (3.1)	0
Instrumental and procedural	Management of drainage	40 (14)	15 (5)	5 (2)	0
	Application of anticoagulants	145 (50)	5 (2)	0	0
	Management of urinary catheter	25 (9)	6 (2)	3 (1)	0
	Techniques for controlling pain	78 (27)	12 (4)	10 (3)	0
	Care involving colostomy bag and barrier	35 (12)	5 (2)	0	0
	Physical activity (physical exercise. walking. travel)	30 (10)	45 (16)	70 (24)	5 (2)
Enjoyment	Management of sadness and anxiety	0	5 (2)	30 (10)	15 (5)
Anticipation	Signs and symptoms of surgical wound infection	80 (28)	100 (34)	20 (7)	0
	Signs and symptoms of urinary tract infection	10 (3.4)	12 (4.1)	13 (4.5)	0
	Permanent telephone hotline	40 (14)	60 (21)	30 (10)	20 (7)
	Administrative processes for authorizing procedures and appointments	40 (14)	120 (41)	40 (14)	0
Social relationships	Sharing with loved ones	30 (10)	60 (21)	25 (9)	15 (5)

Source: Research data (2018)

The global analysis by cancer type and surgical intervention found that the needs for knowledge about diet, eating, and constipation management were presented more frequently in gastrectomized patients. In participants who required prostatectomy, the need related to the management of the bladder catheter was found. Additionally, the need for guidance on care for colostomies in those who underwent surgical intervention for colectomies was found, as was the need for guidance for the detection of signs and symptoms of urinary tract infection (anticipation dimension) in those who required a prostatectomy or hysterectomy.

Regardless of the type of oncological surgery performed, there was a predominant need for the application of anticoagulants, the management of pain techniques, knowledge about diet and eating, physical activity, administrative processes for authorizing procedures and appointments, the permanent telephone hotline, and sharing with loved ones (Table 3).

**Table 3:** Skills required for at-home care of patients, by type of cancer

Dimension	Need	Type of cancer							
		Breast	Stomach	Prostate	Cervical	Ovarian	Lung	Colon and rectum	Other
		(%)	(%)	(%)	(%)	(%)	(%)	(%)	(%)
Knowledge	Diet and eating	6.2	75		29	14	31	44	22
	Constipation		50	8.8				22	25
Uniqueness	Life plan	2.5	15	6				9	26
Instrumental	Management of drainage	75							
	Application of anticoagulants	37.5	25	29	21	29		44	87
	Management of urinary catheter			100					
	Care involving colostomy bag and barrier							89	
	Techniques for controlling pain	25	75	15			31	22	58
	Physical activity	35	60		29	14.3	15	67	87
Enjoyment	Management of sadness and anxiety	10	30		21			13	39
Anticipation	Signs and symptoms of urinary tract infection			44	21				
	Signs and symptoms of surgical wound infection	87.5	50					62	72
	Permanent telephone hotline	18.7	75	35	71	43	31	67	51
	Administrative processes for authorizing procedures and appointments	50	60	50	43	57	46	44	61
Relationships	Sharing with loved ones	25	40	44	64	29	23	22	32

Source: Research data (2018)

## Discussion

The present study demonstrates the multiple home care needs presented by cancer patients who have undergone surgical intervention. In this sense, the role of the oncology nurse is central in this type of follow-up, and communication skills become the focus of the intervention. Effective communication [7] can make the difference in aspects such as the patient’s understanding of the disease and emotional well-being as well as psychological adjustment required by the patient after experiencing the complexity and uncertainty involved in undergoing surgical oncological intervention.

Even though a person with cancer is a reflective human being who is able to make rational decisions, this ability is dependent on contextual aspects such as the conversational frameworks in meetings with the health team. These contextual elements are generally controlled by professionals, and consultation and monitoring can be used to build relationships that facilitate communication and patient support for him or her to take control of the situation [8].

Cancer patients have needs for competencies and skills that persisted throughout all of the telephone follow-ups, a finding that contrasts with the relevance of intervention programs throughout the process that should be initiated from the process of diagnosis, surgery, postoperative care, and recovery, with the establishment of structured guidelines that include the skill components for care with the purpose of training the patient [9].

It should be noted that surgical care is an area in which a large quantity of information is transferred, and patient learning often has to occur within a short period of time. On the other hand, the presence of anxiety and distress becomes a variable that affects the learning process [10], thus requiring the active presence of the nursing team, which modulates and facilitates these types of situations and acts as a key educator in care. Currently, at the hospital, patients obtain education and skills regarding diet, anticoagulation, wound drainage, and ostomy management.

In the first follow-up, high needs were identified in the knowledge, instrumentation, and anticipation dimensions. These findings coincide with other studies that highlight informational needs for pain management, postoperative personal care, and wound healing [11, 12], as well as knowledge of the surgical technique, symptoms, pain medications, thromboprophylaxis (patients are mostly sent home with subcutaneous anticoagulant), mobilization, nutrition, intestinal function (ostomy team, contacts, and administration), possible postoperative complications, and suture removal [13, 14].

During the second and third follow-ups, an increase in the needs for guidance on physical activity, the signs and symptoms of surgical wound infection, the administrative processes for the authorization of procedures and appointments, and sharing with loved ones were noted. It can be inferred that in the second week of the postoperative period, the patient improves his or her physical functionality and, therefore, is interested in strengthening his or her mobility and resuming daily activities. On the other hand, postsurgical medical control, and possible referral to other specialists are needed, implying that information on access to the social security system is essential. It should be noted that in the local context, administrative issues in accessing medical controls and appointments take on special relevance; thus, they should be included among the issues addressed in discharge programs with the management of other professionals such as social workers. A study in patients with chronic diseases conducted in the local context similarly reflected this need, which became a central focus worrying not only patients but their families [15].

The third and fourth follow-ups had an increase in the needs to manage sadness and anxiety (enjoyment dimension) and share with loved ones (relationships dimension). These findings suggest the importance of covering emotional support for these patients. It is likely that in this part of the process, there are emerging concerns about the impact of the surgical intervention, the complementary treatment with chemotherapy and/or radiotherapy, possible changes in daily life, and so forth. In this regard, Archer et al. [16] state that there should be an exploration of psychological disorders and changes that may arise in the postsurgical period because they may alter the patient’s quality of life. Psychosocial factors need to be explored, such as coping styles, the support made available to the patient, and the form in which stressful events are handled.

Regardless of the type of oncological surgery, the dominant needs are for knowledge about diet, eating, and constipation management, with the nutritional pattern and elimination being essential aspects to be discussed in planning the discharge of this type of patient.

Constipation is a common problem in patients with cancer, ranging in incidence from 39 to 70% [17], and it can affect quality of life in all dimensions, including economically due to readmission and the costs of drugs associated with management. It is of imperative importance to benefit from adequate guidance on valuation and management as a key element of follow-up. Trigger factors must be determined, such as certain medications (opioids for pain), lack of physical activity, the type of surgical intervention, and emotional components, among others.

The management of diet and eating is another central topic in follow-up planning, and this finding is similar to that reported regarding patients with chronic diseases [15] and gastrectomized patients [18] in studies carried out in the local context. The education provided must be focused on and oriented towards the needs of the patient [10], in accordance with the type of surgery and the possible restrictions on certain foods, with options and alternatives provided in accordance with the resources that are available. It should be noted that this issue, i.e., the type of food, quantity, restrictions, and specific instructions, often tends to be one of the most worrisome concerns for the family.

On the other hand, it is difficult to overstate the importance of out-of-hospital follow-up programs using information and communications technology (ICT) such as the telephone, taking into account the need indicated by the participants to have access to a permanent line of communication with the healthcare team. Telephone interventions can provide easier access to healthcare and are considered a convenient method for providing support to patients [19]. In addition, the relevance of telephone calls to guide patients is highlighted, including guidance on technical aspects of postoperative management. It is possible that educational and training efforts offered in the hospital scenario will be enhanced with telephone follow-up and other types of technological support, such as videos, to reinforce these efforts.

Based on the patient-centric model, the focus on needs, perspectives, and experiences is crucial. In oncology patients, this type of intervention is thought to contribute to monitoring and managing side effects, to provide psycho-educational care, and to be highly acceptable and accessible for patients [19]. The challenges that arise include some patients’ preferences for conventional, face-to-face consultations, and the length of calls to avoid tedious definition of the process and result indicators.

## Limitations of the Study

The present study contributes to consolidate a follow-up program for patients with cancer who have undergone surgery on the basis of having determined a profile of needs for acquiring skills in at-home care. Despite this important contribution, it is not without from limitations.

First, the conclusions outlined here refer to a specific group of patients from a single institution. Therefore, they must be interpreted with caution, since generalization of results to other contexts is not possible under the design followed up in the present study.

A main conclusion was that patients and families expressed the need for training and guidance on the manipulation of anticoagulants, techniques for controlling pain, management of constipation, and knowledge about diet and physical activity. Nonetheless, these aspects of care may differ among the different cancer types. The large heterogeneity in our sample impeded a comparative analysis that would bring further conclusions. Future studies must be focused on specific types of cancer. For instance, diet and eating was a common concern in the group of gastrectomized patients. This seems to be a promising avenue for future research.

Finally, approaches considering the acceptability, utility, and pertinence of telephone intervention and assistance for discharged patients are required. Specific studies oriented to measure these components of post-surgical care can also compare the alternative use of new technologies (such as video calls or online training).

## Conclusions

Cancer patients who have undergone surgery report needs in the dimensions of skills in at-home care during the telephone follow-up carried out during release. The knowledge, instrumentation, and anticipation dimensions have the greatest incidence in the first and second follow-ups. The third and fourth follow-ups continued to have persistent needs in the anticipation dimension, with an increase in relationship and interaction needs as well as those related to enjoyment.

## Implications for Practice

Telephone follow-up interventions need to be consolidated in hospital release or hospital discharge programs that address these care needs. This study reveals the importance of including follow-up and educational intervention strategies in the training of nurses. Fundamentally, it is necessary to enhance the abilities of nurses to communicate effectively with patients according to an appropriate identification of their particular needs. Finally, consultation with a nurse needs to be incorporated into surgical procedures in Colombia; consultation can take place on-site or by phone calls, and it constitutes a main success factor during the follow-up phase.
